# Advances in Continuous Microfluidics-Based Technologies for the Study of HIV Infection

**DOI:** 10.3390/v12090982

**Published:** 2020-09-04

**Authors:** Joëlle Eid, Marylène Mougel, Marius Socol

**Affiliations:** Institute of Research in Infectious Diseases (IRIM), Centre National de la Recherche Scientifique (CNRS), UMR9004, University of Montpellier, Team R2D2, 34293 Montpellier, France; joelle.eid@irim.cnrs.fr

**Keywords:** microfluidics, HIV, infection, diagnosis, life cycle, replication, retrovirus

## Abstract

HIV-1 is the causative agent of acquired immunodeficiency syndrome (AIDS). It affects millions of people worldwide and the pandemic persists despite the implementation of highly active antiretroviral therapy. A wide spectrum of techniques has been implemented in order to diagnose and monitor AIDS progression over the years. Besides the conventional approaches, microfluidics has provided useful methods for monitoring HIV-1 infection. In this review, we introduce continuous microfluidics as well as the fabrication and handling of microfluidic chips. We provide a review of the different applications of continuous microfluidics in AIDS diagnosis and progression and in the basic study of the HIV-1 life cycle.

## 1. Continuous Microfluidics

Microfluidics is the science of manipulating and controlling fluids, usually in the range of microliters. Two types of microfluidics can be distinguished: continuous microfluidics with the use of channels of tens to hundreds of µm of size, and digital microfluidics that relies on droplets manipulation [1]. Here, we focus on continuous microfluidics, which has been less described than the well-known droplet techniques [2,3]. Due to rapidity, low volumes, precise control, multiplexing, and automation, continuous microfluidics is attractive in a vast spectrum of research works [4,5,6,7]. Microfluidics encompasses the Micro-Total Analysis System known as µ-TAS or “Lab-on-a-Chip” that consists of the integration of multiple functions in a single microfluidic chip. For the choice of material and method in microfluidics applications, it should take into account that the physical concepts depend on scale [8]. For instance, at the microscale, when two fluids from separate reservoirs simultaneously enter a microchannel, they flow parallel to each other without any stirring or mixing [9]. The success of continuous microfluidics relies on its accessibility, customization, and low cost, allowing biologists to design and adapt their own devices to their projects.

**Figure 1:**
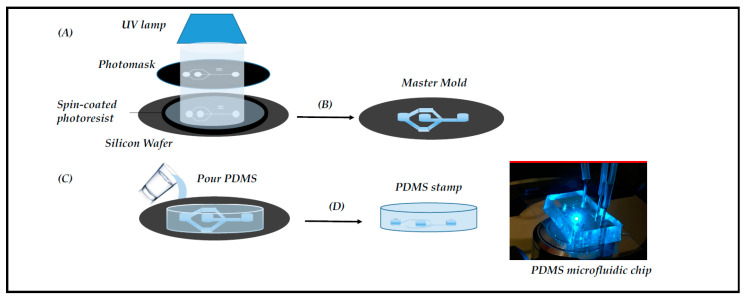
Microfabrication of the chips by photolithography. The main steps of photolithography are: (**A**) Negative SU8 photoresist exposure to UV radiation in order to obtain the pattern drawn on the photomask; (**B**) Photoresistive development to obtain the master mold; (**C**) Pouring the PDMS on the master mold; (**D**) Curing and peeling off the PDMS stamp; finally, making holes in the PDMS for external tubing and binding to a glass slide for microscopy (right picture).

The standard fabrication is the molding method, which encompasses three techniques: replica molding, injection molding, and hot embossing (for review, see [10]). Replica molding, also known as soft lithography, relies on photoactive materials and a polydimethylsiloxane (PDMS) polymer sealed onto either a glass slide, to form a monolayer device, or another PDMS layer to form a multilayer-based system [11] (Figure 1). Additional properties and functions can be added to the devices by integrating membranes, nanowells, or nanopores into the PDMS-based chip for various applications [12,13,14]. The PDMS is transparent, biocompatible, gas-permeable, and elastic. These properties explain why the molding method has been integrated into many biomedical research works [10].

Two other well-known fabrication techniques are the laminate method and 3D printing. The laminate method is defined as the process of manufacturing thermoplastics or optically clear plastics, in multiple layers. The simplicity and rapidity of fabrication make lamination a powerful technique in biological research. Among all of the different techniques for 3D printing, stereolithography is a classic rapid prototyping technique [10]. It is used to create fine features that work through an optical process, built layer on layer. This technology allows the integration of human tissue into a miniaturized device in order to mimic its microenvironment [15].

Besides the traditional microfluidic devices that are fabricated using PDMS or other polymers and plastics, microfluidic devices can also be fashioned from paper. Paper-based devices are especially used in developing countries due to their availability and low cost, since no pump is required for fluid transport (passive transport) [16]. The microfluidic paper-based analytical devices (also called µPADs) are fabricated by patterning many sheets of paper into hydrophilic channels bounded by hydrophobic barriers. Various techniques are available that are summarized in this detailed review [17].

**Figure 2:**
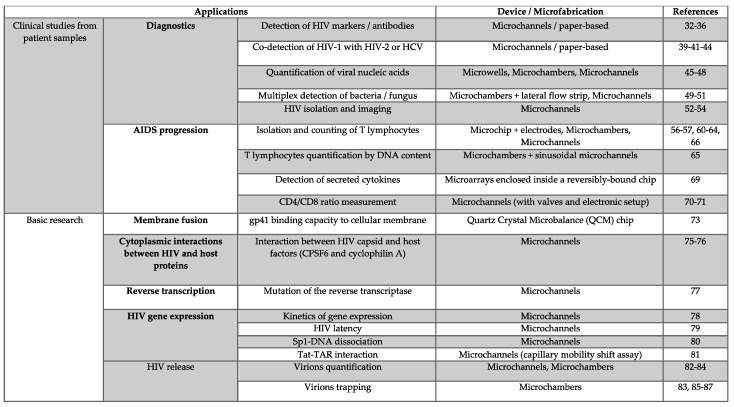
Summary of the different microfluidic devices developed in HIV infection.

In this review, we will describe and discuss the different strategies and devices used in HIV studies in order to facilitate the crucial choice of the most suitable and performant approach, according to one’s need (Figure 2).

## 2. Microfluidics for Studying HIV Infection

**Figure 3:**
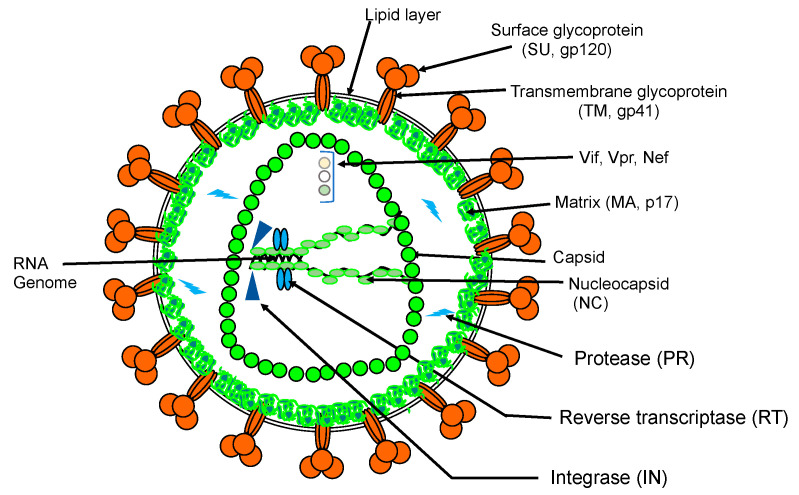
Simplified schematic representation of HIV-1 particle.

HIV-1 induces AIDS and affects several millions of people worldwide. The pandemic persists despite the implementation of highly active antiretroviral therapies [18,19], which must be taken lifelong (for review: [20]). The major causes of HIV-1 resistance to antivirals are due to the high mutation rate of the virus [21] as well as the viral latency with the persistence of silent-HIV reservoirs in infected cells [22]. HIV belongs to the *Retroviridae* family. Its genome is composed of a dimer of two RNA molecules packaged in an enveloped capsid (Figure 3).

HIV enters the cells of the human immune system (especially CD4+ T cells) via the attachment and the fusion of its envelope glycoproteins (Env) with the target cell’s membrane, initiating the infectious cycle. HIV replication cycle lasts approximately 24 h [23,24]. During viral capsid disassembly, the genomic RNA (gRNA) is converted into double-stranded DNA by the viral reverse transcriptase (RT) in a finely regulated timeline (for review: [25]). Interestingly, recent works have reported a nuclear localization of the reverse transcription and the uncoating steps. These data fundamentally change our current spatiotemporal vision of these post entry events [26,27]. Stable integration of this viral DNA into host cell chromosomes by the viral integrase (IN) is an essential step in the life cycle that allows the persistence of viral genomes for the lifespan of infected cells. Viral transcription relies on cellular machinery, which is trans-activated by the viral Tat protein. The primary transcript plays two roles. It ensures viral protein synthesis. HIV-1 uses several non-canonical pathways to translate its own proteins, such as leaky scanning, frameshifting, shunt, and cap-independent mechanisms (for review [28]). Moreover, the viral transcript serves as a viral genome for progeny virions. It forms an RNA dimer in the cytoplasm with the help of the viral Gag protein [29], and the Gag–RNA complex targets the plasma membrane to form new viral particles (for review: [30]). The replication cycle ends with the release of new viral particles, which proceed to the last maturation step to be infectious.

### 2.1. Microfluidic Tools for Diagnostics

Usually, HIV tests are based on the detection of viral antigens or anti-HIV antibodies in the blood by using immunoassays (ELISA, Western blot). HIV can also be sought via its nucleic acids by PCR amplification techniques [31]. A wide range of microfluidic-based systems are currently available that insure a rapid, sensitive, and low-cost diagnosis of HIV infection.

**Figure 4:**
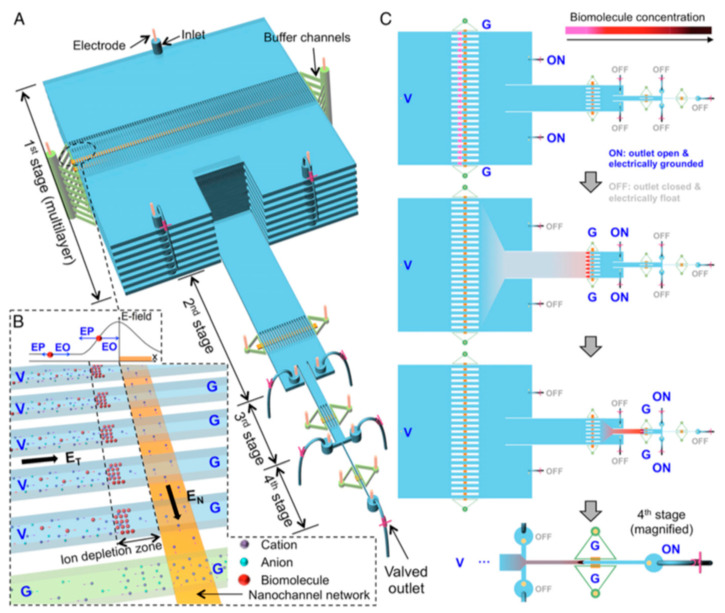
Principle of HOLMES [34]. (**A**) Schematic of HOLMES with hierarchical multistages. At each stage, parallel microchannels and buffer channels are bridged by a thin nanochannel network patterned on the bottom of the microchannels. (**B**) Schematic of nanofluidic biomolecule concentration in massively parallel channels. Under the electrical configuration shown, biomolecules are electro-osmotically injected into the parallel channels and electronically concentrated in the ion deletion zones induced near the micronanochannel junctions. V—voltage; G—grounded; EN and ET—normal and tangential electric fields, respectively; EO—electroosmosis; EP—electrophoresis. (**C**) Schematic of relayed reconcentration of biomolecules from massively parallel microchannels into a single microchannel to dramatically boost the concentration performance.

An automated microfluidic system, named µWestern blot, was developed for the detection of several HIV markers (RT, gp120, p24) and antibodies (anti-gp120/p24). The system unites protein sizing and antibody probing in one system and allows the simultaneous running of 48 µWesterns as well as the analysis of 3 protein targets per blot. Compared to conventional Western blot, the µsystem provides high precision with a low limit of detection (LOD) of 50 pM, a reaction time of 60 min, and 10^3^-fold reduction in reagent consumption [32]. Similarly, several microfluidic devices have been implemented to improve the standard ELISAs used for the detection of p24 protein fluorescence and anti-p24 antibodies from plasma [33,34]. A microtiter technique, called europium nanoparticles-based immunoassay, is miniaturized to a microchip platform. Due to their high photostability and resistance to self-quenching, the europium nanoparticles ensure a stable and high-intensity fluorescence emission, increasing the sensitivity of the system up to attomole levels [33]. Interestingly, a hierarchical nanofluidic molecular enrichment system (namely HOLMES) was developed (Figure 4). Based on electo-osmosis phenomena, it ensures an increase in the biomolecules concentration throughput and speed in ion depletion zones, achieving billion-fold enrichment of biomolecules within 30 min into a single microchannel [34].

There is also another method for the detection of the anti-gp41 marker in blood, which can distinguish different HIV subtypes. This portable and cost-effective immunoassay uses silver reduction. This is performed in a PDMS microchannel network, integrating immunoassay and optical detection. The reduction in silver ions results in a silver film whose opacity is a function of the concentration of the gp41. The system has no photobleaching and the signal is stable for months [35]. Microfluidic immunoassay can also be integrated in paper-based microdevices. A system combining paper-based microdevices and electrochemical impedance spectroscopy detection has shown high sensitivity in p24 detection. It relies on paper-based origami nanobiosensors assembled into two pieces of cellulose paper. This label-free and ultrasensitive detection of p24 antigen has a LOD of 300 fg/mL, which is 33 times lower than that of a commercial p24 ELISA kit (10–15 pg/mL). Additionally, no interference in p24 detection was observed when other virus-associated antigens were present in serum samples [36].

Advances in microfluidic HIV diagnosis systems have encouraged researchers to develop multiplexing devices that allow the detection of co-infections. Multiplexing brings rapidity, high sensitivity, and precision, especially with minimal cross-reactivity. Since hepatitis B and C viruses (HBV, HCV) have a high prevalence in HIV-infected patients and are suspected of HIV-1 co-infection [37,38], it is of great interest to simultaneously diagnose these viruses. Several microfluidic devices have been developed for multiplexed detection which differ by the detection system used [39,40]. One integrates Quantum dots (semiconductor nanoparticles Qdots) into PDMS microchannels fabricated by soft lithography containing sera samples. Qdots are coated with a hepatitis B surface antigen (HBsAg), HCV nonstructural protein 4 (NSP4), and HIV glycoprotein 41 (gp41). Electrokinetics is used to transport Qdots in the microchannels and as a Qdot passes through a focused laser spot, a fluorescence is emitted and detected. The feasibility was demonstrated with the three diagnostic targets: HBV, HCV, and HIV [40].

The paper-based microdevices have also demonstrated advantages for multiplexing with sensitivity and negligible cross-reactivity. The device contains eight electrochemical immunosensors to test multiple serum samples with the simultaneous detection of antibodies against HIV p24 antigen and HCV core antigen [41]. Although HIV-1/HIV-2 co-infection occurs at a low prevalence, it may cause different progression to AIDS than monoinfection and lead to antiretroviral drug resistance [38,42,43]. To detect these two types, a microfluidic barcoded chip was designed that contains several sets of PDMS-based microchannels. HIV-1 anti-gp41/gp120 and HIV-2 anti-gp36 antibodies have been distinguished in six human sera and they appear as dark bars or light spaces, depending on the positive or negative results [44].

A different diagnostic way relies on the detection and the quantification of viral nucleic acids by using quantitative PCR (qPCR). However, the loop-mediated amplification (LAMP) technique is advantageous over standard qPCR, since the use of loop primers accelerates the reaction. It is also cheaper because it does not require expensive thermocyclers. The LAMP technique can be integrated into different microfluidic devices, such as the SlipChip that consists of two plates containing microwells: the top plate loaded with the sample and the bottom plate loaded with the reagents [45]. The performance of the microdevice was compared to that of qRT-LAMP. In the digital format, molecules are separated into compartments and amplified, which requires only an endpoint readout. The initial concentration is determined depending on the number of positive wells, the volume of each well, and the total device volume. However, in a kinetic format, the analyte is amplified in a bulk culture and the amplification progress is monitored as a function of time. Then, the original concentration is determined by comparing the reaction trace to standard curves from solutions of known concentrations. Unlike qRT-LAMP, the device showed high precision due to its ability to distinguish different concentrations of HIV-1 RNA with changes of 3 °C in temperature [45]. The LAMP assay was tested, as well, in a biomolecular amplification reader coupled with a microfluidic cartridge containing 96 reaction chambers. The device showed rapidity in HIV integrase gene amplification detected using calcein metal indicator fluorophore [46]. Nucleic acid quantification is done, as well, with a conventional PCR integrated into microfluidic chips. PCR chips have been considered as a powerful tool for genetic analysis. Usually, the detection of the PCR product is performed by capillary electrophoresis (CE) and fluorescence analysis. However, cross-contamination concerns prevent their reuse. This challenge has been overcome by developing a reusable chip. The reusability of the PCR chambers is ensured by stripping and re-silanizing of the glass surfaces. The system has been validated by the amplification of a 115 bp fragment from the HIV *gag* region [47]. Note that microfluidic systems based on centrifugation-assisted precipitation also have interesting features for nucleic acid detection. One relies on loading the mix of target DNA and GelRed intercalating dye into a microfluidic channel followed by the centrifugation of DNA-GelRed flakes to form a quantifiable precipitate. This approach does not require DNA purification and has been successful in HIV genome quantification with a LOD of 10 ng/µL [48].

**Figure 5:**
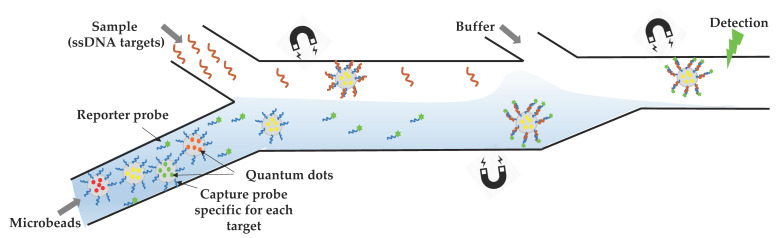
Overall design of the QD barcode assay. Electrokinetics is used to transport QD-barcoded microbeads (Ø = 3.8 µm) into a microchannel. The flow is going from left to right: (i) magnetic barcodes are attracted by a first magnet to interact with the target ssDNA present in the upper stream, (ii) then they move toward the second magnet to interact with the reporter probe present in the lower stream, and (iii) they are pulled by the last magnet into a washing buffer and aligned for optical excitation and detection. They are individually detected by fluorescence as they pass through a focused laser spot. The figure shows the detection of one genetic target, but for multiplexed detection, the same process is done for the different ssDNA targets simultaneously [50].

Since opportunistic infections are more frequent or more severe for immunosuppressed HIV-infected persons (https://aidsinfo.nih.gov/guidelines), it is of great interest to develop multiplexed microsystems to analyze bacterial, fungal, and viral nucleic acids from a single sample. The challenge consists of developing a microfluidic “lab-on-a-chip” that harbors several interrelated components, each miniaturizing one step of the procedure from sample collection to PCR amplification of several pathogens together. For this purpose, a hydraulically driven chip was fabricated, integrating several structures (chambers, membrane, lateral flow strip) into a cassette. After receiving the fluid sample and metering its volume, the multifunctional chip proceeds to a cascade of reactions that occur in different compartments: pathogens lysis, nucleic acids isolation, PCR, and RT-PCR performance. The system showed ability to detect amplicons for both pathogens with 3-fold more sensitivity than the conventional ethidium bromide-stained agarose gels [49]. A simple system with a different detection strategy was successfully able to detect four genetic targets for HIV, HBV, and syphilis (*Treponema pallidum)* [50] (Figure 5). It is based on the injection of Qdot-barcoded beads into a microchannel fabricated by soft lithography and their handling by magnetic force. Despite the sensitivity of the device and the absence of a cross-reaction, it requires off-chip sample pretreatment (cell lysis and nucleic acid extraction) [50].

Besides its implication and efficiency in the detection of HIV nucleic acid, the LAMP technique can also be used for codetection of different pathogens. In this context, a multiple µLAMP was carried out in a microdevice with the shape of a five-pointed star containing five microchannels coated with specific DNA probes for detection of different pathogens. Five different PCR reactions can be done simultaneously. The patient sample and the different reagents are injected via the center hole and are spread evenly through each branch by capillary force. After reaction, the chip is sealed and immersed in a water-bath. The final results were determined by the appearance of green fluorescence induced by SYBR green intercalating dye. This simple and fast technique has been successfully used for the simultaneous detection of HIV-1, tuberculosis bacilli, and *Pneumocystis jirovecii*, known as *Pneumocystis carinii* in saliva [51].

Virus isolation and imaging is another strategy for HIV diagnostics. A whole blood sample of the HIV-infected patient is injected in a microchannel coated with biotinylated anti-gp120 antibody. Viruses are recognized and captured by the antibody, and virus identification is realized by a dual staining, which increases the sensitivity of the device. The system is then improved by designing three microchannels in parallel instead of one: one for sample testing, and two for positive and negative controls. In both cases, the system allowed not only a rapid capturing of HIV particles within 10 min but also a continuous and long-term imaging of the viruses due to the Qdots that provide remarkable photostability and brightness. By immobilizing the Protein G on-chip, it is possible to capture HIV-1 subtypes A, B, and C from culture supernatant or whole blood with viral loads in the range of 10^3^ to 10^5^ copies/mL [52,53,54]. These studies pave the way for a sensitive monitoring of viral load and could replace real-time PCR-based approaches.

### 2.2. Microfluidic Tools for Monitoring AIDS Progression

**Figure 6:**
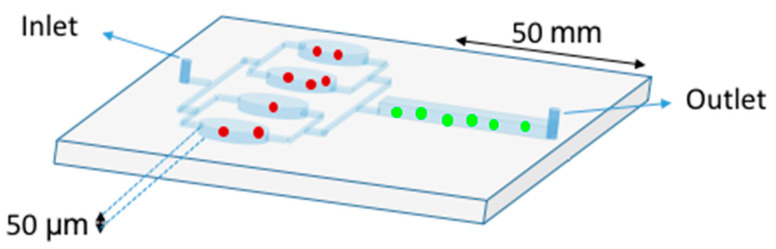
Double Stage Cascade Device used for CD4 counting. The PDMS device harbors two distinct functions: monocyte depletion (upstream) and CD4+ T cell capture (downstream). The upstream region contains four parallel chambers (50 µm height) coated with an anti-CD14 antibody in order to specifically retain the monocytes (red spots). The monocyte depletion increases the sensitivity and specificity of CD4+ T cell (green spots) retention in the main channel (downstream), which was functionalized with an anti-CD4 antibody. After sample injection, rinsing steps are crucial to avoid shearing off captured cells [57].

Identification of the AIDS staging evolution of patients is crucial in the treatment decision. The progression of HIV infection can be monitored by the isolation and the enumeration of CD4+ T lymphocytes in blood samples, which are the primary target cells of HIV. Clinically, a CD4 count below 200 cells/µL establishes the diagnosis of AIDS, and in most settings, is used to initiate antiretroviral treatment and prophylaxis against opportunistic infections [www.who.int/hiv/pub/guidelines/patientmonitoring.pdf]. The most employed technique to detect and to measure physical and chemical characteristics of a population of cells is flow cytometry (FCM). This technique is routinely used in basic research, clinical practice, and trials for cell sorting. FCM relies, among other physical properties, on hydrodynamic focusing in a flow chamber [55]. Another single-cell technique is impedance FCM. This new system is a label-free technology, which has recently been enhanced by the “lab-on-a-chip” approach [56]. It consists of a chip equipped with microelectrodes that measure impedance changes of the medium when cells or other particles pass through the electric field. The absence of optical components reduces setup times and maintenance to a minimum. However, the cytometers are voluminous and expensive. To circumvent these inconveniences, a label-free system was reported [57] (Figure 6). CD4+ T cells and monocytes are distinguished by their different responses to shear stress on the functionalized device surface. Indeed, with a shear stress of 0.2 Pa, the CD4 + T cells were preferentially captured, while the monocytes adhere in minority. This is mainly because monocytes express about one order of magnitude of CD4+ less than the lymphocytes [58,59]. In this study, the retained cells are counted with a simple light microscope. Sensitivity and specificity of these microchip assays for the clinical threshold of 200 cells/µL indicate R2 higher than 0.86 when compared with FCM.

**Figure 7:**
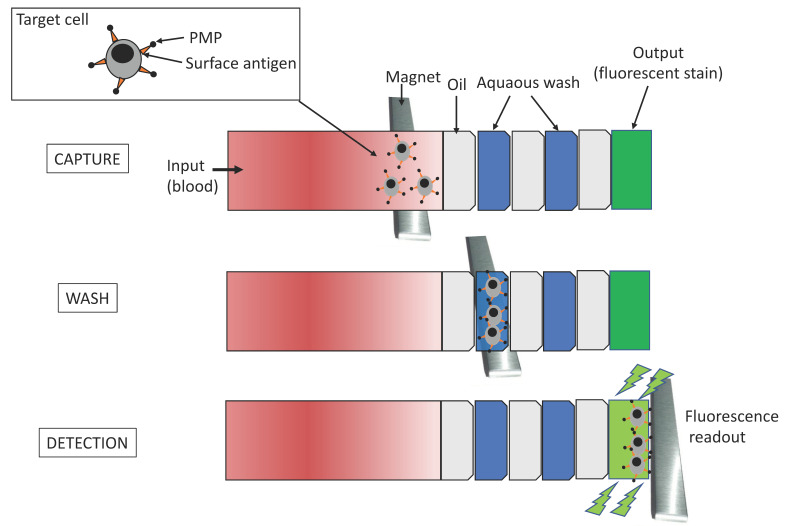
Schematic representation of the IFAST device. It is formed by seven successive flow chambers containing three oil barriers. The magnet at the bottom of the device is moved from the input to the output wells at a rate of 1–2 mm per second. The paramagnetic particles (PMP) move with the magnet, carrying along attached cells and excluding unbound cells at the immiscible phase barrier. Isolated cells are dyed on-chip with a small molecule dye (Calcein AM) metabolized to a fluorophore to be read by a fluorometer [64].

To overcome challenges like chip fabrication and rapid detection and counting of CD4+ T cells, a new integrated platform was developed [60]. CD4+ T immobilization followed the protocol previously described and the captured cells were detected using a charge coupled device (CCD) sensor by the lensless imaging platform: the white light, emitted by a halogen lamp passes through the polymethyl methacrylate cover, reaches the cells, and then, the light intensity of a cell shadow image is determined by diffraction. Further, an automatic cell counting software was developed to enumerate the captured cells in three seconds and the overall process is carried out within 10 min. The integrated platform achieved 83.5 ± 2.4% performance (*n* = 9 devices) compared to FCM. The great performance of this system has conducted to a portable version of the system, which was successfully tested in the USA and Tanzania [61]. The system operates without antibody-based fluorescent labeling and expensive fluorescence microscopy. Qdots [62] or magnetic beads can be associated with cells immunocapturing (immunomagnetic isolation) assays, in which cells are separated by centrifugo-magnetophoresis [63]. This “lab-on-a-disc” contains the PDMS microfluidic channels and the co-rotating magnets. Whole blood is incubated with paramagnetic microparticles that specifically bind phenotypic markers on target cells. This multiforce separation is continuous and its efficiency was up to 92% for cells expressing the HIV/AIDS relevant epitope (CD4) [63]. Meanwhile, an immiscible filtration assisted by a surface tension (IFAST) device was implemented for isolating CD4+ T lymphocytes from whole blood samples [64]. IFAST is a sample preparation method that utilizes water/oil microfluidic interfaces in a PDMS chip to permit rapid magnetic bead-based isolation of cells (Figure 7). The Dynal T4 Quant kit protocol (Invitrogen), which requires a laborious manual data collection process, was modified such that both monocyte depletion and CD4 isolation occur in a pair of IFAST devices.

It is also possible to quantify CD4+ T cells by measuring the DNA content of isolated cells [65]. In this case, magnetic-based immunocapture and DNA-beads assay are integrated into a two-stage PDMS chip. Different masses of silica-coated magnetic beads were added and aggregation between beads and DNA corresponds to the DNA quantity and therefore, the CD4 count. The device achieved an excellent correlation (R^2^ = 0.98) with FCM.

Recently, a new technique of chip microfabrication based on the Inkjet printing method has been adapted to CD4+ T cells trapping [66]. Furthermore, by the same technique, hydrogel layers with embedded fluorophore-labeled antibodies were deposited on polymethyl methacrylate slides onto microfluidic chips. The hydrogel layer maturation consists of controlling antibody release from printed layers for intense and homogeneous on-chip cell staining. After incubation, the chambers are imaged in a custom-built, wide field-of-view fluorescence imager, which also performs the automated image analysis, to count CD4+ T cells (number of cells per μL of blood). Again, this technique showed an excellent agreement with FCM (R^2^ = 0.97; *n* = 57) [66].

To diagnose opportunistic viral or bacterial infections, the ratio of CD4+ and CD8+ T cells or the quantification of the cell-secreted cytokines (such as IL-2 and IFN-γ) are relevant parameters of immune response [67,68]. To this end, printed microarrays of cell- and cytokine-specific antibodies spots were enclosed inside a PDMS reversibly bound chip in order to capture T-cell subsets of red blood cell-depleted whole blood. Mitogenic activation of T-cells followed by immunofluorescent staining of the microarrays revealed fluorescence signals due to binding of IL-2 and IFN-γ on anti-cytokine antibody spots. The cytokine signal was quantified with a microarray scanner after removing the PDMS from the glass slide. [69]. Another strategy for CD4/CD8 ratio measurement is based on creating two independent affinity regions in a single microfluidic channel. Two different monoclonal antibodies are coated separately onto different capture regions. Only 2 μL of lysed human blood samples were loaded to fill the entire channel capacity, followed by a stop flow incubation that selectively captured CD4+ and CD8+ T lymphocytes on each corresponding region. The ratio measurement was achieved within 1h and it showed a close agreement with FCM (R^2^ = 0.97) [70]. A more complex design incremented by a biosensor [71] also harbors a good score compared to FCM (R^2^ = 0.92). The advantage of this biochip is that it can be adapted to enumerate other specific cell types.

### 2.3. Microfluidic Applications in HIV-1 Basic Research

Microfluidics also provides advanced technologies to decipher the molecular mechanisms involved throughout the HIV life cycle, from virus entry, reverse transcription, and gene expression to virus production, mainly at the single cell and single molecule levels.

Upon HIV binding to the host cell receptor and coreceptor, the viral envelop gp120 and the transmembrane gp41 glycoproteins play an essential role in the fusion between virus and cellular membranes, leading to virus entry [72]. In this process, gp41 plays a critical role, since the rate of fusion depends on its fusogenic activity and its expression level. The study of gp41 function may provide new clues in antiviral strategies, since gp41 is a major target. A sensitive method to study gp41 binding capacities has been developed based on epitope-imprinting strategy. A quartz crystal microbalance measures the change in frequency of a quartz crystal resonator. In this study, a synthetic peptide of gp41 was used as a template for molecular imprinting and a microchip was fabricated to bind gp41 epitope by a biomimetic sensor. The system is also able to recognize the whole gp41 proteins with high affinity and selectivity among other biomolecules (Kd = 3.17 nM) [73].

After virus entry, the HIV capsid comes into play. The capsid is a multifunctional protein that coordinates nuclear import, reverse transcription, virus uncoating, and integration [74]. Thus, it interacts with crucial host factors that regulate infection. To study the role of the capsid during these early steps of infection, another type of biosensing strategy has been developed, measuring dual-color fluorescence by total internal reflection fluorescence videomicroscopy. Binding events are observed in real-time on a sensor surface assembled on a glass coverslip located at the bottom of a PDMS chip device. In this study, the sensor surface is coated with antibodies for immobilization of fluorescent self-assembled capsids. The stability of the surface allows its reuse. Binding and dissociation with a fluorescent capsid-partner is detected by the appearance and disappearance of the fluorescent signal colocalized with capsid proteins. Thus, the system measures binding affinity, stoichiometry, and kinetics of host proteins known to interact with the HIV-1 capsid to promote infection. Several capsid partners are already well-known and the proof of concept of the system was realized with the cellular cyclophilin A and CPSF6 (phenylalanine-glycine motifs of cleavage and polyadenylation specificity factor subunit 6) proteins. The biosensor assays showed detection limits for weak interactors with Kd = 1–100 µM (such as CPSF6). This system could accelerate characterization of novel capsid binders [75,76].

The crucial reverse transcription of the HIV genome was the first to be successfully targeted among antiviral strategies. However, the efficiency of anti-RT drug therapy is limited by the emergence of RT mutations that confer HIV resistance to anti-RT compounds. Compared to conventional methods such as gel electrophoresis and melting curve analysis, the use of microfluidics provides compelling advantages in the detection of these mutations. As shown by an in vitro assay developed to discriminate the well-known synthetic K103N point mutation from wild-type template, the technique brings rapidity (30 min), small volumes (5 µL), specificity, and sensitivity in the nanomolar range (100 nM) [77]. The assay includes ligation of specific fluorescent probes to target sequences, duplex denaturation, magnetic microfluidic separation, and fluorogenic detection. The PDMS chip consists in a microchannel 30 mm long tapered to concentrate circulating reagents. A magnet is placed underneath the chip and only the target templates with the magnetic ligation products flow along the channel and will be collected in the outlet well to be detected by a fluorometer. In the future, this technique could be used to assess other mutations that would be responsible for virus resistance to antiviral drugs.

Microfluidic applications are also found in the study of further HIV expression steps. Analysis of intracellular HIV expression often requires cell capture and immobilization in order to probe dynamic processes at the single-cell level. Microfabricated PDMS devices offer an ingenious approach to long-term single-cell studies. Indeed, it is possible to measure single-cell gene-expression kinetics for up to 60 h, as demonstrated by Razooky et al. [78]. They loaded resting primary CD4+ T lymphocytes into a round microwell with a diameter comparable to that of cells. Then, the cells progress into cell-imaging channels by gravity. The cells expressed a GFP-HIV construct, allowing tracking of HIV expression over time, since the device allows rapid and simple media exchange without the displacement of cells [78]. The proposed devices enable experiments with any other cell types. A simple method to achieve cell capture is the insertion of physical traps embedded in the microchannel of PDMS chip connected with inlet and outlet reservoirs. In this case, no pump or tubing is used to aid fluid flow within the device. Suspension cells (Jurkat) are trapped as they passively flow from the inlet to the outlet. The experimental model used with this device consists in a stable clonal cell line, integrating a latent GFP-HIV which requires transcription activation to turn green. To study HIV activation, the chip is fixed on a fluorescence microscopy stand and time-lapse images are taken for up to 24 h. Activation was stimulated by transcription factor activators or histone deacetylase inhibitors. Interestingly, they achieve latent HIV activation with different and uncorrelated rates and onset times. Thus, these results reveal the complexity of the reactivation mechanisms, moderating the effectiveness of the clinical “activate-and-kill” strategy [79].

Intracellular molecular mechanisms have been more closely investigated by using microfluidics, as exemplified by this study of the cellular transcription factor, Sp1, which regulates HIV transcription [80]. To do so, a binding assay platform was constructed that implements fluorescence correlation spectroscopy (FCS) on a PDMS titration chip to measure the binding of Sp1 to a HIV DNA promoter. The chip harbors a serpentine channel for mixing the reagents and after opening a valve, the mixture enters a nanoliter-sized analysis chamber. The chamber can be isolated by closing the valves to stop the flow in the microchannel. Then, fluorescently labeled free DNA and DNA bound to Sp1 are quantified by FCS in a purely diffusion-governed environment. The system also enables titration experiments of an intercalative drug that dissociates Sp1–DNA interaction. FCS results confirm that Sp1 bound strongly to the GGGAGG sequence and that the intercalative drug tested (doxorubicin) has a IC_50_ = 0.55 µM [80], comparable to previously published values. Different microfluidic systems have been developed to study protein–nucleic acid interactions based on capillary mobility shift assays incorporated in a microfluidic chip. The authors chose the well-characterized system of the binding of HIV trans-activator of transcription (Tat) to the transactivation-responsive RNA (TAR). Tat/TAR interaction enhances elongation transcription, resulting in productive transcription of the HIV full-length genome. Briefly, samples containing short peptides and oligoribonucleotides are injected into microchannels and are separated by pressure-driven flow and through the application of an electric potential difference. Molecules and/or complexes are subsequently visualized via LED-induced fluorescence. It is also possible to use full-length Tat proteins instead of short peptides. Like the above system using FCS on a PDMS titration chip, this setup is applicable to examining drug impact on the interaction in a dose-dependent manner. Here, the authors calculated an IC_50_ for the neomycin inhibitor = 1.6–3 µM [81]. Compared to FCS-based microfluidics, this method presents the advantage of performing assays in high-throughput screening-compatible formats. These two microfluidic-based methods are versatile tools to study any types of molecular bindings.

The last step of the HIV life cycle corresponds to the release of the virus progeny into the extracellular medium. Rapid quantification of viral particles in solution by flow virometry has been an elusive goal for virologists. Microfluidics provides several answers to this long-lasting need. Most methods use fluorescently labeled HIV (GFP-HIV) or HIV bound on fluorescent beads to distinguish viral particles from extracellular exosomes, since both two nanoparticles share similar range of sizes. First of all, it is important to know that similar virus production kinetics are obtained when culturing cells in PDMS microdevices under continuous flow or in a standard tissue culture in plates. In addition, viral titers are independent of the shear stress that cells undergo in the chip [82]. The main challenge for flow virometry systems is the specificity and the sensitivity of the signals, which must correspond to individual virus detection in culture medium or physiological samples (<100 µL). Optical tweezers, which use the momentum of photons to trap viruses, achieve promising results [83]. HIV GFP virions are delivered into a microfluidic chamber at the concentration of 0.4–4.0 × 10^8^ virions/mL. They are immediately detected by infrared laser due to the two-photon excitation of GFP by the trapping laser. This optical trapping virometry has single-molecule resolution and can also discriminate between individual and viral aggregates, since they produced different laser deflection signals [83]. A commercial apparatus is now available for nanoparticle tracking analysis that allows virions quantification with a LOD—1.7 × 10^7^ particles/mL [84]. However, better LOD can be reached by complexifying the microdevices while keeping the sample processing as a simple flow-through process. For instance, enhancement of viral detection is obtained by the fabrication of porous devices with a nanopore array over flatbed microchannels [85]. The system was tested with biotinylated HIV virions captured in channels with surface functionalized with Avidin (NeutrAvidin). High HIV capture yields (80%) are reached for a large range of HIV concentrations (10^3^–10^6^ virions/mL) [85]. A clever biosensing system has also been proposed that consists in electrical sensing viruses through capacitance spectroscopy on a flexible plastic chip with printed electrodes. The advantage of this system is its low LOD with reliable measurement of 10^2^ virions/mL. However, the method requires several off-chip steps including: capture of virions by magnetic beads conjugated with biotinylated anti-gp120 antibodies, extended washing to remove residual electrically conductive backgrounds, and lysis of captured virions by Triton x-100 before injection into the chip for virions measurement [86,87]. Therefore, this system cannot be used for dynamic studies. Actually, to date, there are no relevant techniques for kinetics study of viral production. This is mainly due to the LOD limitation of the current techniques. The implementation of new microfluidic strategies to study virus release in real-time from a single-cell will constitute the next challenge in the field of retrovirology.

## 3. Conclusions

Microfluidics has revolutionized the fields of biology and health by offering the possibility of managing biological samples in tiny channels and chambers. Microfluidic technology is a powerful tool with many advantages such as: rapidity, reliability, specificity, multiplexing, and inexpensiveness. It also allows studies at the single-cell scale and the development of high-throughput approaches.

Microfluidics has recently entered the field of HIV-1. This review tackles continuous microfluidics and their different implications in the study of HIV infection. This information is summarized in Figure 2 in order to help the investigator to choose the most appropriate technique to use. Although this review has been written with the prototypic HIV-1 retrovirus in mind, the techniques are described in order to benefit to a larger community.

## References

[1] Agarwal A. (2013). Digital microfluidics: Techniques, their applications and advantages. J. Bioeng. Biomed. Sci..

[2] Chacon O.L.A., Baret J.C. (2019). Rapid stabilization of droplets by particles in microfluidics: Role of droplet formation. Chem. Syst. Chem..

[3] Mashaghi S., Abbaspourrad A., Weitz D.A., van Oijen A.M. (2016). Droplet microfluidics: A tool for biology, chemistry and nanotechnology. TrAC Trends Anal. Chem..

[4] Malbec R., Chami B., Aeschbach L., Ruiz B.G.A., Socol M., Joseph P., Leichlé T., Trofimenko E., Bancaud A., Dion V. (2019). μ LAS: Sizing of expanded trinucleotide repeats with femtomolar sensitivity in less than 5 minutes. Sci. Rep..

[5] Sackmann E.K., Fulton A.L., Beebe D.J. (2014). The present and future role of microfluidics in biomedical research. Nature.

[6] Socol M., Ranchon H., Chami B., Lesage A., Victor J.M., Bancaud A. (2019). Contraction and tumbling dynamics of DNA in shear flows under confinement induced by transverse viscoelastic forces. Macromolecules.

[7] Socol M., Wang R., Jost D., Carrivain P., Vaillant C., Le Cam E., Dahirel V., Normand C., Bystricky K., Victor J.-M. (2019). Rouse model with transient intramolecular contacts on a timescale of seconds recapitulates folding and fluctuation of yeast chromosomes. Nucleic Acids Res..

[8] Bazant M.Z., Squires T.M. (2004). Induced-charge electrokinetic phenomena: Theory and microfluidic applications. Phys. Rev. Lett..

[9] Whitesides G.M. (2006). The origins and the future of microfluidics. Nature.

[10] Gale B., Jafek A., Lambert C., Goenner B., Moghimifam H., Nze U., Kamarapu S.K. (2018). A review of current methods in microfluidic device fabrication and future commercialization prospects. Inventions.

[11] Hamon M., Hong J.W. (2013). New tools and new biology: Recent miniaturized systems for molecular and cellular biology. Mol. Cells.

[12] De Jong J., Lammertink R.G., Wessling M. (2006). Membranes and microfluidics: A review. Lab Chip.

[13] Rettig J.R., Folch A. (2005). Large-scale single-cell trapping and imaging using microwell arrays. Anal. Chem..

[14] Rudenko M.I., Holmes M.R., Ermolenko D.N., Lunt E.J., Gerhardt S., Noller H.F., Deamer D.W., Hawkins A., Schmidt H. (2011). Controlled gating and electrical detection of single 50S ribosomal subunits through a solid-state nanopore in a microfluidic chip. Biosens. Bioelectron..

[15] Jang J., Park J.Y., Gao G., Cho D.W. (2018). Biomaterials-based 3D cell printing for next-generation therapeutics and diagnostics. Biomaterials.

[16] Gong M.M., Sinton D. (2017). Turning the page: Advancing paper-based microfluidics for broad diagnostic application. Chem. Rev..

[17] Li X., Ballerini D.R., Shen W. (2012). A perspective on paper-based microfluidics: Current status and future trends. Biomicrofluidics.

[18] Margolis D.M., Archin N.M., Cohen M.S., Eron J.J., Ferrari G., Garcia J.V., Gay C.L., Goonetilleke N., Joseph S.B., Swanstrom R. (2020). Curing HIV: Seeking to target and clear persistent infection. Cell.

[19] Mocroft A., Vella S., Benfield T.L., Chiesi A., Miller V., Gargalianos P., d’Arminio M.A., Yust I., Bruun J.N., Phillips A.N. (1998). Changing patterns of mortality across Europe in patients infected with HIV-1. Lancet.

[20] Chupradit K., Moonmuang S., Nangola S., Kitidee K., Yasamut U., Mougel M., Tayapiwatana C. (2017). Current peptide and protein candidates challenging HIV therapy beyond the vaccine era. Viruses.

[21] Yeo J.Y., Goh G.R., Su C.T., Gan S.K. (2020). The determination of HIV-1 RT mutation rate, its possible allosteric effects, and its implications on drug resistance. Viruses.

[22] Cohn L.B., Chomont N., Deeks S.G. (2020). The biology of the HIV-1 latent reservoir and implications for cure strategies. Cell Host Microbe.

[23] Holmes M., Zhang F., Bieniasz P.D. (2015). Single-cell and single-cycle analysis of HIV-1 replication. PLoS Pathog..

[24] Mohammadi P., Desfarges S., Bartha I., Joos B., Zangger N., Munoz M., Günthard H.F., Beerenwinkel N., Telenti A., Ciuffi A. (2013). 24 hours in the life of HIV-1 in a T cell line. PLoS Pathog..

[25] Mougel M., Houzet L., Darlix J.L. (2009). When is it time for reverse transcription to start and go?. Retrovirology.

[26] Burdick R.C., Li C., Munshi M., Rawson J.M.O., Nagashima K., Hu W.S., Pathak V.K. (2020). HIV-1 uncoats in the nucleus near sites of integration. Proc. Natl. Acad. Sci. USA.

[27] Dharan A., Bachmann N., Talley S., Zwikelmaier V., Campbell E.M. (2020). Nuclear pore blockade reveals that HIV-1 completes reverse transcription and uncoating in the nucleus. Nat. Microbiol..

[28] Guerrero S., Batisse J., Libre C., Bernacchi S., Marquet R., Paillart J.C. (2015). HIV-1 replication and the cellular eukaryotic translation apparatus. Viruses.

[29] Ferrer M., Clerte C., Chamontin C., Basyuk E., Laine S., Hottin J., Bertrand E., Margeat E., Mougel M. (2016). Imaging HIV-1 RNA dimerization in cells by multicolor super-resolution and fluctuation microscopies. Nucleic Acids Res..

[30] Mailler E., Bernacchi S., Marquet R., Paillart J.C., Vivet-Boudou V., Smyth R.P. (2016). The life-cycle of the HIV-1 gag-RNA complex. Viruses.

[31] Hurt C.B., Nelson J.A.E., Hightow-Weidman L.B., Miller W.C. (2017). Selecting an HIV test: A narrative review for clinicians and researchers. Sex. Transm. Dis..

[32] Hughes A.J., Herr A.E. (2012). Microfluidic western blotting. Proc. Natl. Acad. Sci. USA.

[33] Liu J., Du B., Zhang P., Haleyurgirisetty M., Zhao J., Ragupathy V., Lee S., De Voe D.L., Hewlett I.K. (2014). Development of a microchip Europium nanoparticle immunoassay for sensitive point-of-care HIV detection. Biosens. Bioelectron..

[34] Ouyang W., Han J. (2019). Universal amplification-free molecular diagnostics by billion-fold hierarchical nanofluidic concentration. Proc. Natl. Acad. Sci. USA.

[35] Sia S.K., Linder V., Parviz B.A., Siegel A., Whitesides G.M. (2004). An integrated approach to a portable and low-cost immunoassay for resource-poor settings. Angew. Chem. Int. Ed..

[36] Li X., Liu X. (2016). A microfluidic paper-based origami nanobiosensor for label-free, ultrasensitive immunoassays. Adv. Healthc. Mater..

[37] Chew K.W., Bhattacharya D. (2016). Virologic and immunologic aspects of HIV-hepatitis C virus coinfection. AIDS.

[38] Requena S., Caballero E., Lozano A.B., Rios-Villegas M.J., Benito R., Rojo S., Cabezas T., Macià M.D., Nieto M.D.C., Soriano V. (2019). Treatment outcome in dually HIV-1 and HIV-2 coinfected patients living in Spain. AIDS.

[39] Corstjens P.L.A.M., Chen Z., Zuiderwijk M., Bau H.H., Abrams W.R., Malamud D., Niedbala R.S., Tanke H.J. (2007). Rapid assay format for multiplex detection of humoral immune responses to infectious disease pathogens (HIV, HCV, and TB). Ann. N. Y. Acad. Sci..

[40] Klostranec J.M., Xiang Q., Farcas G.A., Lee J.A., Rhee A., Lafferty E.I., Perrault S.D., Kain K.C., Chan W.C.W. (2007). Convergence of quantum dot barcodes with microfluidics and signal processing for multiplexed high-throughput infectious disease diagnostics. Nano Lett..

[41] Zhao C., Liu X. (2016). A portable paper-based microfluidic platform for multiplexed electrochemical detection of human immunodeficiency virus and hepatitis C virus antibodies in serum. Biomicrofluidics.

[42] Mendoza C., Lozano A.B., Caballero E., Cabezas T., Ramos J.M., Soriano V. (2020). Antiretroviral therapy for HIV-2 infection in non-endemic regions. AIDS Rev..

[43] Nsagha D.S., Njunda A.L., Kamga H.L., Assob J.C., Bongkem E.A. (2012). HIV-1/HIV-2 co-infection among voluntary counselling and testing subjects at a regional hospital in Cameroon. Afr. Health Sci..

[44] Zhang Y., Sun J., Zou Y., Chen W., Zhang W., Xi J.J., Jiang X. (2015). Barcoded microchips for biomolecular assays. Anal. Chem..

[45] Selck D.A., Karymov M.A., Sun B., Ismagilov R.F. (2013). Increased robustness of single-molecule counting with microfluidics, digital isothermal amplification, and a mobile phone versus real-time kinetic measurements. Anal. Chem..

[46] Myers F.B., Henrikson R.H., Bone J., Lee L.P. (2013). A handheld point-of-care genomic diagnostic system. PLoS ONE.

[47] Prakash R., Kaler K.V.I.S. (2007). An integrated genetic analysis microfluidic platform with valves and a PCR chip reusability method to avoid contamination. Microfluidics Nanofluidics.

[48] Banerjee I., Aralaguppe S.G., Lapins N., Zhang W., Kazemzadeh A., Sonnerborg A., Neogi U., Russom A. (2019). Microfluidic centrifugation assisted precipitation based DNA quantification. Lab Chip.

[49] Abrams W.R., Barber C.A., McCann K., Tong G., Chen Z., Mauk M.G., Neogi U., Russom A. (2007). Development of a microfluidic device for detection of pathogens in oral samples using upconverting phosphor technology (UPT). Ann. N. Y. Acad. Sci..

[50] Gao Y., Lam A.W., Chan W.C. (2013). Automating quantum dot barcode assays using microfluidics and magnetism for the development of a point-of-care device. ACS Appl. Mater. Interfaces.

[51] Xu L., Kong J. (2013). A multiplexed nucleic acid microsystem for point-of-care detection of HIV co-infection with MTB and PCP. Talanta.

[52] Kim Y.G., Moon S., Kuritzkes D.R., Demirci U. (2009). Quantum dot-based HIV capture and imaging in a microfluidic channel. Biosens. Bioelectron..

[53] Wang S., Esfahani M., Gurkan U.A., Inci F., Kuritzkes D.R., Demirci U. (2012). Efficient on-chip isolation of HIV subtypes. Lab Chip.

[54] Wang S., Ip A., Xu F., Giguel F.F., Moon S., Akay A., Kuritzkes D.R., Demirci U. (2010). Development of a microfluidic system for measuring HIV-1 viral load. Proc. SPIE Int. Soc. Opt. Eng..

[55] Cossarizza A., Chang H.D., Radbruch A., Acs A., Adam D., Adam-Klages S., Agace W.W., Aghaeepour N., Akdis M., Allez M. (2017). Guidelines for the use of flow cytometry and cell sorting in immunological studies. Eur. J. Immunol..

[56] Cheung K., Gawad S., Renaud P. (2005). Impedance spectroscopy flow cytometry: On-chip label-free cell differentiation. Cytom. A J. Int. Soc. Anal. Cytol..

[57] Cheng X., Gupta A., Chen C., Tompkins R.G., Rodriguez W., Toner M. (2009). Enhancing the performance of a point-of-care CD4+ T-cell counting microchip through monocyte depletion for HIV/AIDS diagnostics. Lab Chip.

[58] Murphy F.J., Reen D.J. (1996). Differential expression of function-related antigens on newborn and adult monocyte subpopulations. Immunology.

[59] Wintergerst E.S., Jelk J., Asmis R. (1998). Differential expression of CD14, CD36 and the LDL receptor on human monocyte-derived macrophages. A novel cell culture system to study macrophage differentiation and heterogeneity. Histochem. Cell Biol..

[60] Moon S., Gurkan U.A., Blander J., Fawzi W.W., Aboud S., Mugusi F., Kuritzkes D.R., Demirci U. (2011). Enumeration of CD4+ T-cells using a portable microchip count platform in Tanzanian HIV-infected patients. PLoS ONE.

[61] Moon S., Keles H.O., Ozcan A., Khademhosseini A., Haeggstrom E., Kuritzkes D., Demirci U. (2009). Integrating microfluidics and lensless imaging for point-of-care testing. Biosens. Bioelectron..

[62] Jokerst J.V., Floriano P.N., Christodoulides N., Simmons G.W., McDevitt J.T. (2008). Integration of semiconductor quantum dots into nano-bio-chip systems for enumeration of CD4+ T cell counts at the point-of-need. Lab Chip.

[63] Glynn M., Kirby D., Chung D., Kinahan D.J., Kijanka G., Ducree J. (2014). Centrifugo-magnetophoretic purification of CD4+ cells from whole blood toward future HIV/AIDS point-of-care applications. J. Lab. Autom..

[64] Howard A.L., Pezzi H.M., Beebe D.J., Berry S.M. (2014). Exclusion-based capture and enumeration of CD4+ T cells from whole blood for low-resource settings. J. Lab. Autom..

[65] Liu Q., Chernish A., Du V.J.A., Ouyang Y., Li J., Qian Q., Bazydlo L.A.L., Haverstick D.M., Landers J.P. (2016). The ARTμS: A novel microfluidic CD4+ T-cell enumeration system for monitoring antiretroviral therapy in HIV patients. Lab Chip.

[66] Wasserberg D., Zhang X., Breukers C., Connell B.J., Baeten E., van Blink D., Benet E.S., Bloem A.C., Nijhuis M., Wensing A.M.J. (2018). All-printed cell counting chambers with on-chip sample preparation for point-of-care CD4 counting. Biosens. Bioelectron..

[67] Pantaleo G., Harari A. (2006). Functional signatures in antiviral T-cell immunity for monitoring virus-associated diseases. Nat. Rev. Immunol..

[68] Pantaleo G., Koup R.A. (2004). Correlates of immune protection in HIV-1 infection: What we know, what we don’t know, what we should know. Nat. Med..

[69] Zhu H., Stybayeva G., Macal M., Ramanculov E., George M.D., Dandekar S., Revzin A. (2008). A microdevice for multiplexed detection of T-cell-secreted cytokines. Lab Chip.

[70] Li W., Gao Y., Pappas D. (2015). A complementary method to CD4 counting: Measurement of CD4+/CD8+ T lymphocyte ratio in a tandem affinity microfluidic system. Biomed. Microdevices.

[71] Hassan U., Watkins N.N., Reddy B., Damhorst G., Bashir R. (2016). Microfluidic differential immunocapture biochip for specific leukocyte counting. Nat. Protoc..

[72] Chen B. (2019). Molecular mechanism of HIV-1 entry. Trends Microbiol..

[73] Lu C.H., Zhang Y., Tang S.F., Fang Z.B., Yang H.H., Chen X., Chen G.-N. (2012). Sensing HIV related protein using epitope imprinted hydrophilic polymer coated quartz crystal microbalance. Biosens. Bioelectron..

[74] Novikova M., Zhang Y., Freed E.O., Peng K. (2019). Multiple roles of HIV-1 capsid during the virus replication cycle. Virol. Sin..

[75] Lau D., Walsh J.C., Peng W., Shah V.B., Turville S., Jacques D.A., Bocking T. (2019). Fluorescence biosensor for real-time interaction dynamics of host proteins with HIV-1 capsid tubes. ACS Appl. Mater. Interfaces.

[76] Peng W., Shi J., Marquez C.L., Lau D., Walsh J., Faysal K.M.R., Byeon C.H., Byeon I.-J.L., Aiken C., Bocking T. (2019). Functional analysis of the secondary HIV-1 capsid binding site in the host protein cyclophilin A. Retrovirology.

[77] Zhang L., Wang J., Roebelen J., Tripathi A. (2015). A simple microfluidic assay for the detection of ligation product. Mol. Diagn. Ther..

[78] Razooky B.S., Gutierrez E., Terry V.H., Spina C.A., Groisman A., Weinberger L.S. (2012). Microwell devices with finger-like channels for long-term imaging of HIV-1 expression kinetics in primary human lymphocytes. Lab Chip.

[79] Ramji R., Wong V.C., Chavali A.K., Gearhart L.M., Miller-Jensen K. (2015). A passive-flow microfluidic device for imaging latent HIV activation dynamics in single T cells. Integr. Biol..

[80] Yeh H.C., Puleo C.M., Lim T.C., Ho Y.P., Giza P.E., Huang R.C., Wang T.-H. (2006). A microfluidic-FCS platform for investigation on the dissociation of Sp1-DNA complex by doxorubicin. Nucleic Acids Res..

[81] Fourtounis J., Falgueyret J.P., Sayegh C.E. (2011). Assessing protein-RNA interactions using microfluidic capillary mobility shift assays. Anal. Biochem..

[82] Vu H.N., Li Y., Casali M., Irimia D., Megeed Z., Yarmush M.L. (2008). A microfluidic bioreactor for increased active retrovirus output. Lab Chip.

[83] Pang Y., Song H., Kim J.H., Hou X., Cheng W. (2014). Optical trapping of individual human immunodeficiency viruses in culture fluid reveals heterogeneity with single-molecule resolution. Nat. Nanotechnol..

[84] Steppert P., Burgstaller D., Klausberger M., Tover A., Berger E., Jungbauer A. (2017). Quantification and characterization of virus-like particles by size-exclusion chromatography and nanoparticle tracking analysis. J. Chromatogr. A.

[85] Surawathanawises K., Kundrod K., Cheng X. (2016). Microfluidic devices with templated regular macroporous structures for HIV viral capture. Analyst.

[86] Shafiee H., Jahangir M., Inci F., Wang S., Willenbrecht R.B., Giguel F.F., Tsibris A.M.N., Kuritzkes D.R., Demirci U. (2013). Acute on-chip HIV detection through label-free electrical sensing of viral nano-lysate. Small.

[87] Shafiee H., Kanakasabapathy M.K., Juillard F., Keser M., Sadasivam M., Yuksekkaya M., Hanhauser E., Henrich T.J., Kuritzkes D.R., Kaye K.M. (2015). Printed flexible plastic microchip for viral load measurement through quantitative detection of viruses in plasma and saliva. Sci. Rep..

